# HTS-Driven Discovery of New Chemotypes with West Nile Virus Inhibitory Activity

**DOI:** 10.3390/molecules15031690

**Published:** 2010-03-12

**Authors:** Dong Hoon Chung, Colleen B. Jonsson, Clinton Maddox, Sara N. McKellip, Blake. P. Moore, Marintha Heil, E. Lucile White, Subramaniam Ananthan, Qianjun Li, Shuang Feng, Lynn Rasmussen

**Affiliations:** 1 Department of Biochemistry and Molecular Biology, Southern Research Institute, 2000 9th Ave S. Birmingham, AL 35205, USA; E-Mails: cbjons01@louisville.edu (C.B.J.); maddox@southernresearch.org (C.M.); mckellip@southernresearch.org (S.N.M.); b.moore@southernresearch.org (B.P.M.); white@southernresearch.org (E.L.W.); Ananthan@southernresearch.org (S.A.); feng@southernresearch.org (S.F.); rasmussen@southernresearch.org (L.R.); 2 Center for Predictive Medicine for Biodefense and Emerging Infectious Diseases, Department of Microbiology and Immunology, 505. S. Hancock, University of Louisville, KY 40222, USA; 3 Southern Research Institute, 431 Aviation Way, Frederick, MD 21701, USA; E-Mail: HEIL@southernresearch.org (M.H.); 4 Division of Infectious Disease, 1900 University Boulevard, THT 229, University of Alabama at Birmingham, AL 35294, USA; E-Mail: liq@uab.edu (Q.L.)

**Keywords:** West Nile virus, high throughput screen, antivirals, chemotypes

## Abstract

West Nile virus (WNV) is a positive sense, single-stranded RNA virus that can cause illness in humans when transmitted via mosquito vectors. Unfortunately, no antivirals or vaccines are currently available, and therefore efficient and safe antivirals are urgently needed. We developed a high throughput screen to discover small molecule probes that inhibit virus infection of Vero E6 cells. A primary screen of a 13,001 compound library at a 10 μM final concentration was conducted using the 384-well format. Z′ values ranged from 0.54–0.83 with a median of 0.74. Average S/B was 17 and S/N for each plate ranged from 10.8 to 23.9. Twenty-six compounds showed a dose response in the HT screen and were further evaluated in a time of addition assay and in a titer reduction assay. Seven compounds showed potential as small molecule probes directed at WNV. The hit rate from the primary screen was 0.185% (24 compounds out of 13,001 compounds) and from the secondary screens was 0.053% (7 out of 13,001 compounds) respectively.

## 1. Introduction

West Nile virus (WNV) is a positive sense, single-stranded RNA virus and a member of the genus flaviviruses, Family *Flaviviridae*. In nature, birds are the sylvatic reservoir for WNV in an endemic cycle with the primary vector, the mosquito. The virus is wide spread in Africa, the Middle east and Russia [1], and since the 1999 outbreak in New York [2,3], WNV has spread from east to west in most states in North America [4]. Transmission of the virus to humans via mosquitoes can cause significant health problems such as West Nile fever and a neuroinvasive disease [5,6,7]. Unfortunately, no antivirals or vaccines are currently available, and therefore efficient and safe antivirals are urgently needed. 

The 11 Kb genome of WNV has a single, open reading frame, which is translated into one polypeptide (in the order:C-prM-E-NS1-NS2A-NS2B-NS3-NS4A-NS4B-NS5 [8]) and processed into 3 structural and 7 non-structural proteins. The viral NS3 protease mediates post-translational modification of the polyprotein [9,10] in the cytoplasm and by host proteases in the endoplasmic reticulum. WNV replicates in various types of cells in tissue culture, including Vero E6 [11], BHK, and insect cell lines. During replication, host cells may show a cytopathic effect (CPE) from apoptosis [12]. WNV infection can induce apoptosis in several different cell lines, including, insect cells and mammalian cells, possibly through the *bax* gene [13]. 

Development of assays and high throughput (HT) screening platforms for WNV has followed a path similar to a number of other viruses with a focus on cell-based replicon reporter assays and biochemical assays. WNV replicons have been created which harbor luciferase or GFP alone [14] or replicons with luciferase and Neo^r^ reporters [15]. The replicons were adapted to a 96-well format, and the assay was validated with known inhibitors of WNV [16,17]. The first HT screen of WNV consisted of diverse set of 200 compounds in which triaryl pyrazoline was identified as an inhibitor of viral RNA synthesis [17]. In another study, a small library of 108 compound pyrolizines were identified which may inhibit RNA synthesis [18]. In 2006, Gu *et al.*, published a screen of a library of over 35,000 small molecule compounds with the WNV replicon with the luciferase and Neo^r^ reporters [16,19]. They also developed a counterscreen in a 96-well format with live virus which was employed to screen 23 compounds. From these screens, several candidate compounds were identified with one class, a pyrazolopyrimidine, showing promising activity and selectivity as well as promise for follow-up chemistry.

We have developed a cell based assay using WNV (NY-99 strain) in 384-well format that builds from our prior success with live viral HT screens for the influenza virus and SARS CoV [20,21]. The assay employed cell viability as the end point, using Promega’s CellTiter-Glo®, which produces a luminescent signal in relation to the quantity of ATP in host cells (directly related to cell viability). In contrast to dye formation or dye-uptake methods, which have a low dynamic range, the CellTiter-Glo® assay shows a higher dynamic range with less background. We implemented the WNV assay in the 384-well format in a screen of 13,001 compounds. A diverse group of small molecules targeting various specific steps in virus replication was discovered in the screening campaign.

## 2. Results and Discussion

### 2.1. Assay optimization and implementation of live WNV for HTS

**Figure 1:**
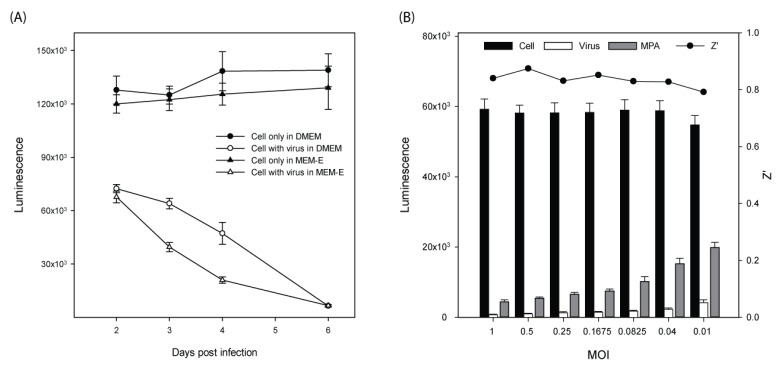
Assay optimization for assay media, incubation period, and MOI. (A) Vero E6 cells were infected with WNV at 0.05 MOI using the 96-well plate format and developed on successive days. Virus cultured in MEM-E produced a higher CPE between day 3 and day 5 than in DMEM. (B) Assay robustness with Z´ factor and inhibition profiles using MPA (positive control) and different amounts of virus in a 384-well plate format. Z´ factor was higher than 0.75 in a 0.01~1 MOI range. The inhibitory activity of MPA was greater at the lower MOI.

To optimize the assay for robust and reproducible HTS data, we explored the assay media, number of cells per well, multiplicity of infection (MOI) and incubation time post-infection. We tested complete DMEM, MEM-E with or without non-essential amino acids (NEAA) and MEM-E with reduced NaHCO_3_ (1.3 g/L) [17]. We did not find a significant difference among the three MEM-E based media with regard to luminescence (data not shown). However, cytopathic effect (CPE) was more evident in cells grown and infected with WNV in MEM-E than in DMEM. The signal from the virus control with DMEM showed a higher luminescence signal than that with MEM-E while the luminescence from cell controls was identical (Figure 1A). The MEM-E media also shortened the assay incubation period by two days compared to DMEM media and hence was chosen for the HTS. 

We tested the MOI from 1.0 to 0.01 in 384-well plates. We did not witness a significant difference in cell viability between different MOIs (Figure 1B). The Z′ value was >0.6 at any MOI tested confirming the development of a very robust assay. The efficacy of the positive control compound, mycophenolic acid (MPA), varied depending on infectious dose. At MOI 0.04, MPA showed 30% protection without decreasing Z′ value. Based on these findings we have developed optimized conditions as described in Materials and Methods. 

### 2.2. Single dose HTS results

**Figure 2:**
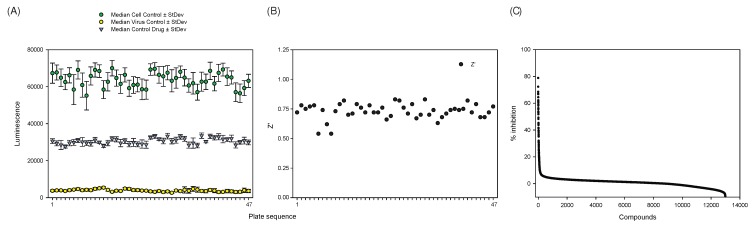
Assay performance and robustness and % inhibition profiles of compounds in the primary HTS. (A) The average luminescence readings for cell control, virus control and control drug, MPA at 5 μg/mL of 47 plates depicted with standard deviations (B) Assay robustness depicted with Z′ values with a median of 0.74. (C) Percent inhibition ranging from 79% to -10% with a mean of 0.70%.

A primary screen of a 13,001 compound library at a 10 μM final concentration was conducted using the 384-well format. Z′ values ranged from 0.54–0.83 with a median of 0.74. Average S/B was 17 and S/N for each plate ranged from 10.8 to 23.9 with a mean of 16.94 and standard deviation of 2.95 (Figure 2A and Figure 2B). These statistical values indicate that the assay provided high quality and the required robustness and reproducibility of an HTS assay. In this assay the overall average inhibitions were 0.7% with a standard deviation of 4.184% and the control drug MPA showed 44.6% inhibition on average. The percent inhibition cutoff was 13.25% that represents the viral mean + three standard deviations, a common method of statistically defining hits in a single dose screen. Using this cutoff method, 13.25%, 92 compounds were identified as active WNV inhibitors (Figure 2C). 

### 2.3. Dose response assay

To confirm the activity and to test the cytotoxicity, the top 110 inhibitory compounds were evaluated in a dose response assay using the identical screen. The secondary assay revealed 24 compounds with a dose dependent response and fifteen of those compounds showed EC_50_ values less than 30 μM. Three compounds, however, were cytotoxic with low CC_50_ values ranging from 16.21 to 36.75 μM. Remaining 21 compounds gave CC_50_ values greater than 50 μM (Table 1). Ten compounds showed an SI_50_ (Selectivity Index 50, ratio of CC_50_ to EC_50_) >5 and these were evaluated further (Table 1). 

**Table 1:** Twenty-four compounds selected from the primary and dose response screening.

Supplier ID	EC_50_	CC_50_	SI_50_
SRI-2176	1.9	16.21	8.53
SRI-10806	6.15	36.75	5.98
SRI-22003	12.18	>100	>8.2
SRI-11928	12.83	23.39	1.82
SRI-795809	13.88	>100	>7.2
SRI-1001309	16.94	>100	>5.9
SRI-16635	18.4	>100	>5.4
SRI-16537	18.64	>100	>5.4
SMR000370276	18.9	>100	>5.3
SRI-1665808	19.58	>100	>5.1
SMR000394098 *	19.6	>100	>5.1
SMR000059052 *	20.01	>100	>5.0
SRI-12784	22.91	>100	>4.4
SRI-12896	23.43	>100	>4.3
SMR000027739 *	29.58	>100	>3.4
SMR000171908	30.35	>100	>3.3
SRI-4578 *	30.39	>100	>3.3
SRI-2967	34.11	>100	>2.9
SRI-18457	34.22	>100	>2.9
SRI-6050	37.34	60.02	1.61
SRI-19093	40.96	>100	>2.4
SRI-6488	41.99	>100	>2.4
SRI-1055	51.58	>100	>1.9
SRI-13037	58.68	>100	>1.7

* Compounds were not available at the time of re-supplying from the suppliers.

### 2.4. Time of addition assay

The compounds selected by the primary and dose response screening were re-supplied from the suppliers as a solid form except 4 compounds; SMR000394098, SMR000059052, SMR000027739 and SRI-4578, which were not available at that time (Table 1). Time of addition assays were conducted in a dose response format with the 20 compounds. Serially diluted compounds were added to Vero E6 cells at -1, 4, 8 and 18 h post-infection. Luminescent data was calculated (as above) to obtain EC_50_ values for each compound at each time point of addition. Seventeen compounds showed EC_50_ values less than 30 μM and 5 compounds were not active.

**Figure 3:**
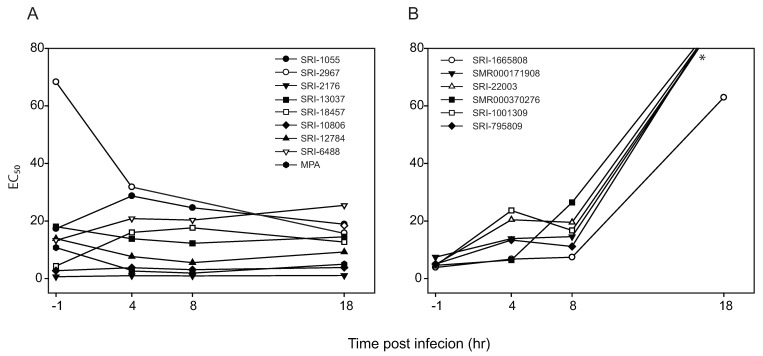
Examples of changes in EC_50_ at different times post-infection. Dose response assay in 384-well format was executed for each time point and the EC_50_ calculated as described in the text. (A) Compounds failed to exhibit significant change in EC_50_ within 18 h post-infection. Meanwhile, the other compounds showed sudden increase in EC_50_ between 8 and 18 h post-infection (B). * EC_50_ calculated was higher than 60 μM. For graphical purpose, the lines were extended beyond border of graph.

Interestingly, some compounds showed no significant change in EC_50_ over the time of addition (Figure 3A). A similar pattern was seen with the positive control (MPA). The EC_50_ of MPA was within a range of 2.0~5.0 μM regardless of when it was added. In contrast, other compounds showed a dramatic change in EC_50_ according to the time of addition (Figure 3B). SRI-19093 showed a minor change (3.2 to 6.4 μM) in EC_50_, and the remaining six compounds in this category showed an EC_50_ value of over 60 μM when the compounds were added 18 h post-infection. The EC_50_ values of the compounds in this class were less than 25 μM when the compounds were delivered prior to 8 h post-infection. We classified the compounds according to the pattern of change in the values of the EC_50_ as shown in Table 2 and Figure 3. 

**Table 2:** The pattern of EC_50_ change depending on the time of addition.

EC_50_ change pattern	Example
No Change : EC50_1hr_ ≈ EC50_4hr_ ≈ EC50_8hr_ ≈ EC50_18hr_	MPA, SRI-12784
Increase: EC50_1hr_ ≈ EC50_4hr_ ≤ EC50_8hr_ < EC50_18hr_	SRI-1665808, SRI-795809, SRI-1001309, SRI-22003, SMR00171908, SMR000370276

EC50_Xhr_ represents the EC_50_ value of the compound when it is administrated at X h post-infection. See Figure 3.

### 2.5. WNV NS2b-NS3pro assay

**Figure 4:**
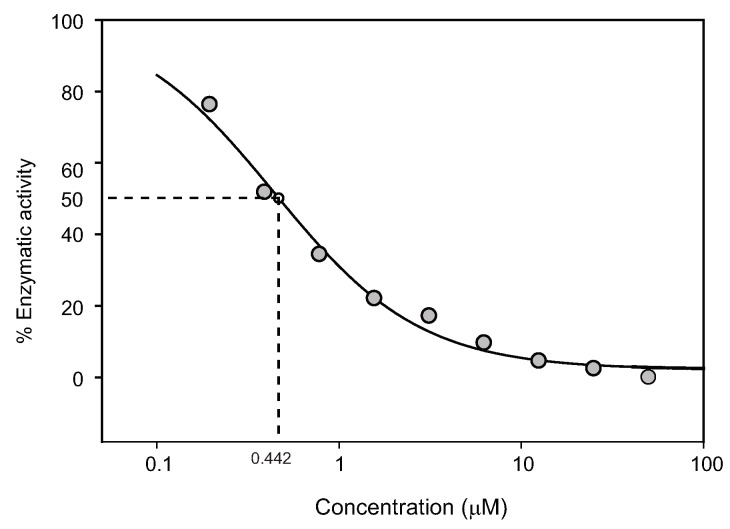
Dose response anti-NS2b-NS3pro activity of SRI-19093. IC_50_, denoted in the figure, was measured as 0.44 μM. Standard Curves, Four Parameter Logistic Curve was used for plotting regression curves and calculating IC_50_ values.

The results from the time of addition assay led us to examine the NS3 protease as a potential target of the hit compounds. NS3 protease is an important enzyme responsible for the maturation of viral proteins at early stage during the replication of virus in the host cells. We tested the compounds for an inhibitory activity on NS3 protease using a FRET based, *in vitro*, enzymatic assay. As a result, we identified SRI-19093 with an inhibitory effect on the protease and with IC_50_ of 0.42 μM, suggesting the molecular target of these two compounds may be the NS3pro of WNV (Figure 4). We also tested N-phenylanthranilic acid for NS3pro inhibitory activity as this moiety was common for two compounds, SRI-19093 and SMR000171908, but we could not detect any significant activity in this system (data not shown).

### 2.6. Titer reduction assay

**Figure 5:**
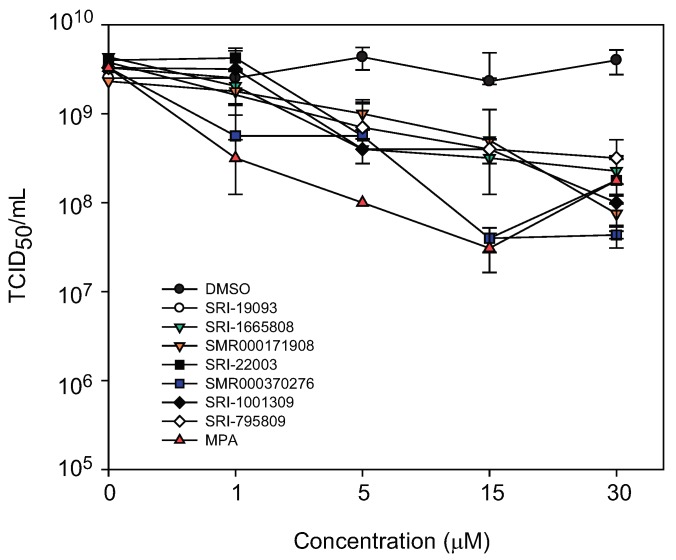
Titer reduction assay in a dose response format. Virus was grown in the presence of compound and then harvested 48 h post-infection. Virus titer was measured as TCID_50_/mL. Each data point represents the mean from experiments performed in duplicate.

**Figure 6:**
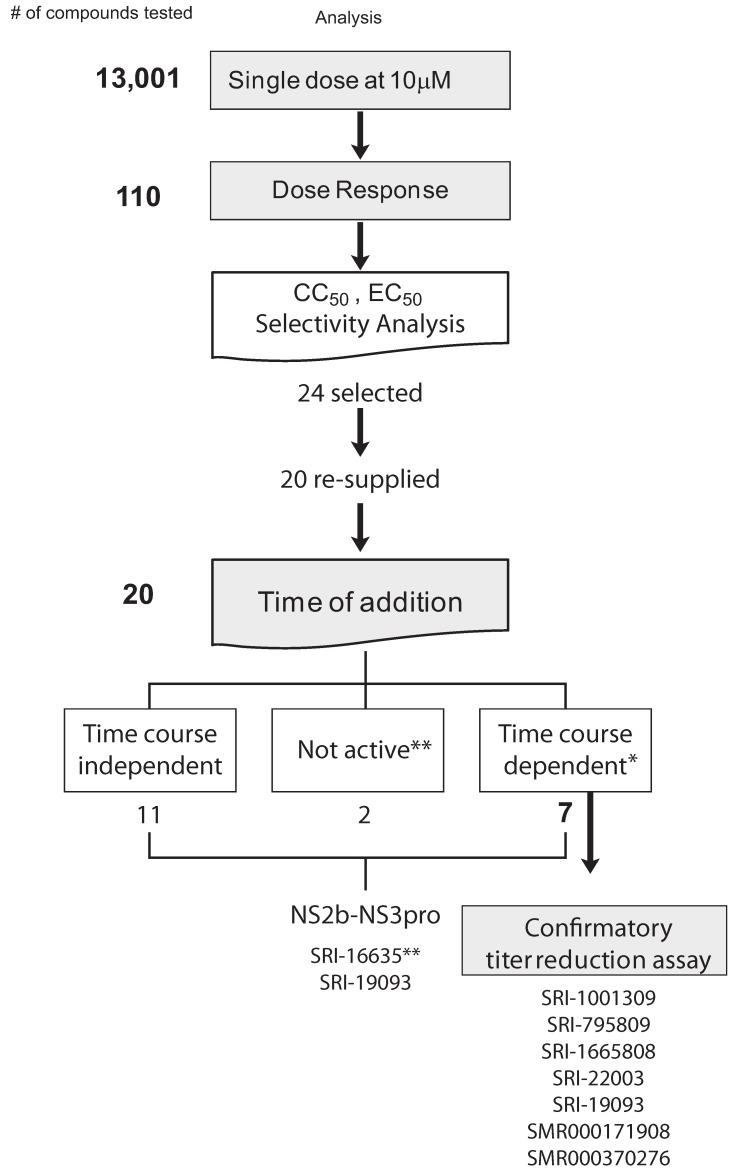
Schematic diagram of discovery for anti-WNV probes from a 13,001 compounds library. The numbers represent the number of compounds tested or categorized.

Finally, we employed a titer reduction assay to confirm the antiviral activity of the seven compounds confirmed in the assays. The titer of the progeny virus produced in the presence of the compounds was measured in TCID_50_ format. Infection of Vero E6 cells using a 0.1 MOI resulted in around 3.3 × 10^9^ TCID_50_/mL in two days post-infection. Treatment of infected cells with the compounds decreased the progeny titer up to 100 fold (Figure 5). For example, SMR000370276 and SRI-22003 showed 98.8% inhibition followed by SRI-19093 and SRI-1665808 at 93.7 and 90.4% inhibition, respectively, at 15 μM concentration. SRI-19093 was most potent in this assay with EC_90_ of 2.14 μM and SRI-7968 was least active with EC_90_ of 26.16 μM. The positive control drug, MPA, worked extremely well as a potent inhibitor with an EC_90_ of 0.94 μM in this assay. The titer reduction assay confirmed the antiviral activity of the hit compounds selected through our pathway (Figure 6).

* All of the compounds with weak correlation in the correlation analysis have turned out to be “replication dependent” in the time of addition assay.
** SRI-16635 was inactive in the time of addition assay (data not shown) but showed anti-NS2b-NS3pro activity in the protease assay.

**Table 3:** Structures and Inhibitory Activities of Selected Compounds.

Compound I.D.	Structure	Dose response in CPE based		Dose response in Titer reduction		SI_50_ *	NS2B-NS3pro	EC_50_ change pattern over the course of infection
		EC_50_ (μM)	CC_50_ (μM)	EC_50_ (μM)	EC_90_ (μM)		EC_50_ (μM)	
**SRI-22003**	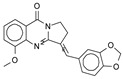	12.18	>30	4.70	5.13	>6	Not active	Increase
**SRI-795809**	Structure not disclosed	13.88	>30	1.14	26.16	>30	Not active	Increase
**SRI-1001309**	Structure not disclosed	16.94	>30	5.46	16.69	>6	Not active	Increase
**SMR000370276**	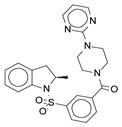	18.9	>30	No solution	6.07	>5	Not active	Increase
**SRI-1665808**	Structure not disclosed	19.58	>30	1.29	7.22	>30	Not active	Increase
**SMR000171908**	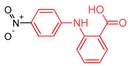	30.35	>30	1.16	6.73	>25	Not active	Increase
**SRI-19093**	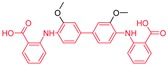	40.96	>30	0.13	2.14	>200	0.44	Increase
**MPA**	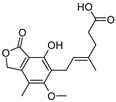		>30	0.03	0.94	>1000	Not active	no change
**N-Phenylanthranilic acid**	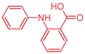	**NT**	NT	NT	**NT**	NT	>50	not active

### 2.7. Discussion 

Herein we report the discovery of several new chemotypes from an HT screen of a 13,001 compound library that inhibit WNV replication (Table 3). The endpoint we developed and employed in the HT screen was CPE caused by viral replication, which has advantages over other target based HTS. A CPE based antiviral screen is expected to elucidate a wider range of antiviral compounds than other target based assays. A CPE based assay is likely to discover cytotoxic compounds since the cytotoxic compounds will decrease the endpoint signal and are read as inactive. This has an advantage over reporter-based assays in that they can read false positives. In fact, we found only 14 compounds with CC_50_ less than 30 μM among 110 compounds subjected to the dose response assay. The pathway for probe discovery is depicted in Figure 7. The top 110 compounds, based on their inhibitory activities were examined by a dose response and cytotoxicity assay. Twenty-four compounds showing a dose response inhibition were further evaluated in a time of addition assay and in a titer reduction assay. Finally, seven compounds are presented as anti-WNV probes. Hit rate for the secondary assay and the final confirmation were 0.185% (24 compounds out of 13,001 compounds) and 0.053% (7 out of 13,001 compounds) respectively.

We categorized the 7 compounds into 3 chemical groups based on their chemical structures (Table 3). The first group are uridine derivatives; SRI-795809, SRI-1665808 and SRI-1001309 (full chemical structure not disclosed). Similar compounds have been known for WNV antiviral activity [22]. The second group is composed of SRI-19093 (Redoxal) and SMR000171908. SRI-19093 was also active against flaviviruses with inhibitory activity on dihydroorotate dehydrogense [23]. The third group of compounds, SRI-22003 and SMR000370276, are new chemotypes with anti-flavivirus activity. SMR000370276 has shown activity in other HTS assays, such as anti-TNF-α-specific NF-kB induction and NOD1 and 2 inhibition assays (AID: 1852, 1578 and 1566 http://pubchem.ncbi.nlm.nih.gov/). All seven compounds screened in the time of addition experiment were confirmed to have anti-WNV activity based on the titer reduction assay and EC_90_ values between 2.14~26.16 μM. This suggests that HTS and following assays are an effective method for probe discovery and evaluation. None of the compounds showed anti-WNV activity as good as the positive control drug, MPA, however, the mechanism of MPA is not specific to the virus making the probes we identified of significant interest. 

Our time of addition analysis showed interesting patterns in the value of EC_50_. These studies suggest possible targets for each compound. We showed that there are two types of patterns in EC_50_ that change according to the time of addition; 1) compounds with minimal change in EC_50_, and 2) the compounds with an increased EC_50_ MPA reflected the first pattern and nucleoside derivatives, SRI-1001309 and SRI-795809 produced the second pattern. This suggests that compounds that do not have a change in their EC_50_ may target the host while those that change, target the virus.

We identified one compound, SRI-19093, that inhibited viral protease in vitro and time of addition studies. These results suggest it might target the viral protease NS2B-NS3pro. SRI-19093 exhibited low activity in the primary, dose response (EC_50_ of 41 μM), and time of addition assays. 

## 3. Experimental

### 3.1. Cells and Virus

All work with WNV was performed at BSL-3 following CDC guidelines. Vero E6 cells (ATCC CRL 1586) were maintained in Dulbecco’s modified Eagle’s medium (DMEM) supplemented with 10% fetal bovine serum (FBS) and 2 mM L-glutamine. WNV strain NY-99 was used for the assays and was propagated in a complete Minimum Essential Medium Eagle (MEM-E) media obtained from Sigma-Aldrich (St. Louis, USA), supplemented with 10% FBS, 100 units/mL penicillin, 100 μG/mL streptomycin and 2 mM L-glutamine. The virus stock was divided into 1 mL aliquots and stored at -80 °C. 

### 3.2. Viral Assay

Virus plaque forming units were measured using an agar overlay method described previously [17] with minor modifications. 

### 3.3. Compound Library Composition and Plating

For primary HT screening, two compound sets were used for a total of 13,001 compounds: the Southern Research Institute (SRI) proprietary compound library composed of 12,343 compounds and a selection of 658 compounds from the National Institutes of Health Molecular Libraries Screening Network library of 200,000 compound that were active in a previous screen against Bluetongue virus (http://pubchem.ncbi.nlm.nih.gov/assay/-assay.cgi?aid=1251&loc=ea_ras/). The SRI proprietary library consists of the historical, unique compounds designed and synthesized at SRI over many years as potential drug candidates. The members of this collection have high biological relevance, particularly to cancer and infectious disease. Major classes of compounds that are represented in this collection include nucleosides, purines, pyrimidines, pteridines, imidazoles, pyridines, quinolines, triazines, disaccharides, guanidines, ureas and carbamates. 

The stock concentrations of both compound sets were 10 mM in 100% DMSO. In the single dose primary screen, the stock compounds were diluted 1:100 in a 10× solution using the Biomek FX giving a 100 μM working concentration in 1% DMSO. A 3 μL aliquot of the working concentration plus an additional 2 μL of assay media was transferred into each well. Control stocks were prepared in assay media at a 6 fold higher than the target concentration: 0.6% DMSO for cell and viral control and 30 μg/mL mycophenolic acid (MPA) at 0.6% DMSO for positive control. MPA was purchased from Sigma-Aldrich Co. (St. Louis, USA) and solubilized in 100% DMSO to a 5 mg/mL stock concentration. Controls were added separately to the assay plate in 5 μL aliquots via the Biomek FX. With the addition of 20 μL of cells and 5 μL of virus, the final test concentration for the compounds was 10 μM and for MPA was 5 μg/mL. The final DMSO was 0.1%, which is well below the 0.5% maximum allowable for this assay. 

After reviewing the data from the primary screen, 110 compounds were selected for a dose-response study. The same compound stocks (10 mM in 100% DMSO) were used to select 7 μL of the compounds into a low-volume, 384-well plate. The dose response plates were prepared using a “stacked-plate” format using a 1:2 dilution series for a total of ten concentrations. By using this format, each entire plate represents one of the ten concentrations in the dilution series. A 5 μL aliquot of each of the serial dilution plates was transferred to the assay plates along with an equal volume but separate transfer of controls. With the addition of 20 μL of cells and 5 μL of virus, the final test concentrations for the dose response screen were 30 μM, 15 μM, 7.5 μM, 3.75 μM, 1.875 μM, 0.9375 μM, 0.4688 μM, 0.2344 μM, 0.1172 μM, and 0.0586 μM all at 0.3% DMSO.

### 3.4. High-Throughput Screen 

Vero E6 cells were suspended in complete MEM-E at 400,000 cells/mL and dispensed at 15 μL into black, clear-bottom, tissue culture treated, 384-well plates (6,000 cells/well) using a Matrix WellMate® (Matrix, ThermoFisher). The plated cells were maintained overnight at 37 °C with 5% CO_2_ in an actively humidified incubator. Test compounds were then transferred at 5 μL per well for a final DMSO concentration of 0.1% (see above). Each plate consisted of 32 wells representing the Cell control, 24 wells for Virus control, 8 wells for positive control (MPA), and 320 wells for testing individual compounds. Cell plates were transferred into the BSL3 and infected by addition of 10 μL of WNV suspension at 2.4 × 10^4^ pfu/mL in complete MEM-E using a Matrix WellMate®. Plates were incubated for 96 h at 37 °C and 5% CO_2_ to promote virus replication. 30 μL of CellTiter-Glo® (Promega, Madison, WI) was added to each well using a Matrix WellMate and incubated at room temperature for 30 min. Luminescence was measured using a Perkin Elmer Envision plate reader (Wellesley, MA) with an integration time of 0.1 s.

### 3.5. Antiviral Efficacy and Cytotoxicity in Dose-Response and Time of Addition Assays

Cytotoxicity and antiviral efficacy were performed in a dose-response format in parallel. Compounds were serially diluted in complete media (MEM-E) with a 0.5% DMSO final concentration and adding 5 μL to cell plates, identical to the stacked plate method [21]. A ten point, two-fold dilution series of compound concentrations ranging from 30 μM to 0.059 μM were used in the assay. Virus (0.04 MOI) or media was added for antiviral effect or cytotoxicity respectively. The plates were incubated 96 h in a 5% CO_2_ incubator at 37 °C. Cell viability was measured as stated above. The serially diluted compounds were also used for time of addition studies adding to the infected cells in 384-well plates at -1, 4, 8 and18 h post-infection with WNV (0.02 MOI). The plates were then developed as above 96 h after infection. The EC_50_ value was calculated for each time of addition point. 

### 3.6. Statistics and Data Analysis

The Z′ value was employed to evaluate the assay’s robustness and was calculated from 1-(3×standard deviation of cell control (σ_c_) + 3* standard deviation of the virus control (σ_v_)/ [mean cell control signal (μ_c_) minus mean virus control signal (μ_v_)]). The signal/background (S/B) was calculated from the mean cell control signal (μ_c_) divided by the mean virus control signal (μ_v_). The signal/noise (S/N) was calculated from mean cell control signal (μ_c_) minus mean virus control signal (μ_v_) divided by the (standard deviation of the cell control signal (σ_c_)^2^ minus the standard deviation of the virus control signal (σ_v_)^2^)^1/2^ [24]. The effective concentration at which the drug inhibited cell death at 50% in the presence of virus (EC_50_) and the cytotoxicity of the drug alone at 50% (CC_50_) were calculated using ActivityBase software (IDBS, Inc, Guildford, UK). CPE inhibition (%) and cell viability were calculated as described in elsewhere [25]. The standard curve analysis function in SigmaPlot™ was used to calculate the inhibitory concentration (IC_50_ and IC_50_ values) for the protease and titer reduction assays. 

### 3.7. WNV NS2b-NS3pro Assay

A recombinant NS3 protease expressed and purified from *E.coli* with the cofactor NS2b (residues 49-96) and a fluorescence resonance energy transfer (FRET) based assay kit were purchased from AnaSpec Inc (CA, USA). We followed an assay protocol published on line at PubChem by University of Pittsburgh Molecular Library Screening Center (http://www.ncbi.nlm.nih.gov/, AID:577). The assay was performed in a 384-well format with 10 ng/well of NS2b-NS3pro enzyme.

### 3.8. Titer Reduction Assay 

Compounds that showed specific inhibitory effects on virus replication steps were further validated for their efficacy using a TCID_50_ reduction assay. Vero E6 cells were seeded in a 12-well plate in a volume of 1 mL and incubated overnight at 37 °C and 5% CO_2_. The next day media was aspirated from the cells and they were infected by adsorption of WNV (0.1 MOI) for one hour in 100 μL media. After aspirating the virus and washing the cells with phosphate buffered saline, media was replenished containing compounds at various concentrations. The plates were incubated for 48 h. Progeny virus titer was measured by TCID_50_ assay in 384-well plate format with 6 wells per dilution of virus. CellTiter-Glo® reagent was employed to determine a cell death which is a sign of infection. A well with a luminescence signal less than the mean of the non-infected control signal, minus 5 times the standard deviation of the control, was regarded as a positive infection. The TCID_50_ was calculated with the numbers of positive infection and negative infection by the Reed-Muench method [26].

## 4. Conclusion 

In summary, we report our approaches in the development and use of an HT screen to discover specific and effective anti-WNV probes. As a result of HTS and secondary assays, we have identified 7 compounds as specific and effective anti-WNV probes. Further optimization of these compounds is needed to develop effective antiviral treatment for WNV infection. In addition, the compounds we have identified should serve as novel molecular probes to help us understand the biology of WNV. Future studies will focus on how these compounds target virus replication and the mechanism of action. In conclusion, identification of previously known and novel moieties from a screening library is proof that the WNV assay is an effective HT screen. 
